# Supplementary material for: Neurocomputational mechanisms involved in adaptation to fluctuating intentions of others
Source: Nat Commun. 2024 Apr 12;15:3189. doi: 10.1038/s41467-024-47491-2 (PMC11014977; doi:10.1038/s41467-024-47491-2)
Supplement: Supplementary file 1 — Supplementary Information [file 41467_2024_47491_MOESM1_ESM.pdf]

Supplementary Information

Supplementary Methods

Instruction for participants

Instructions were in French originally. Below, we added both the English and French versions. Deliberately, we never used words synonymous with “against” or “partner” since such vocabulary could influence the participant’s prior with respect to the goal of the other.

Instructions (english): You are going to be faced with 4 cards: two face down, those of the person you will be interacting with, and two face up, yours. Each turn you will have to choose one of your 2 cards. When the other player has chosen her card, the card will be put in the middle face down without you being able to know which one has been chosen. When both of you have made your choices, you will see the card that the other player has chosen and a one-euro coin if you win, or a crossed-out one-euro coin if you lose. You win if both of you chose the same color card, otherwise you lose. You do not know what are the rules of the game for the person you are interacting with and you do not know which reward she will receive. There will be around 150 trials to perform in the scanner. There will be a one minute break half way through. The person with who you interact will not change between the 2 blocks. Every time you win, you will receive an extra 10 cents as a reward.

Instructions (french): Vous allez être face à 4 cartes : deux faces cachées, celles de la personne avec laquelle vous allez interagir, et deux faces visibles, les vôtres. A chaque tour, vous devrez choisir une de vos 2 cartes. Lorsque l'autre joueur aura choisi sa carte, celle-ci sera posée au milieu, face cachée, sans que vous puissiez savoir laquelle a été choisie. Lorsque vous aurez tous deux fait votre choix, vous verrez la carte que l'autre joueur a choisie et une pièce d'un euro si vous gagnez, ou une pièce d'un euro barrée si vous perdez. Vous gagnez si les deux joueurs ont choisi la même couleur de carte, sinon vous perdez. Vous ne connaissez pas les règles du jeu de la personne avec laquelle vous interagissez et vous ne savez pas quelle récompense elle recevra. Il y aura environ 150 essais à réaliser dans le scanner. Il y aura une pause d'une minute à mi-parcours. La personne avec laquelle vous interagissez ne changera pas entre les 2 blocs. Chaque fois que vous gagnerez, vous recevrez 10 centimes supplémentaires comme récompense.

Debriefing of participants

We debriefed participants and asked the following 3 questions: Did you notice any changes in the way the other player played? If so, which ones? How did you notice it?

At least 16 participants had a sense that the AA was switching between two different strategies (some participants did not answer anything specific to these questions). Below, we translate these debriefing reports:

1. The other player was losing money as he tried to do the "long sequence" technique, but it backfired (nb. The participant seems to think he is playing competitively.). However, sometimes he tried to avoid me.
2. He changes his strategy. In the second game, it was tighter, he understood my strategy.
3. Sometimes the other player would change a move when they were both winning, it looked like he was bored. It looked like the other player was changing.
4. The other had two strategies, either he stayed on the same card while losing, or he changed every other time or tried to change the sequence.
5. Sometimes he would make series of up to 6 times the same card, sometimes he would alternate every two or three choices.
6. What worked at one moment did not work afterwards [...]. We found a logic and then suddenly it didn't work anymore.
7. Sometimes the other would always choose the same card for a long time, then he would vary, then choose the other card all the time.
8. The other person changes his strategy, his intention. At first it looked like a teammate, then an opponent.
9. The other was not consistent. [Sometimes he was consistent, and sometimes he was not consistent.

10. Several changes. When I had the right result, he would change. He would make me feel confident and then change the strategy.
11. He adapted, he made changes in strategy like me.
12. He tried to adapt different strategies by repeating choices or alternating.
13. I noticed changes of rhythm. Sometimes the opponent would follow me and then stop following me. Maybe he was changing the instructions.
14. He would change sequences. Sometimes he would choose 7 times the black and then alternate once of each color [...].
15. At first the other had the same rules, then he changed his strategy (Or did he have reasons to do so?), then sometimes he would go back to the same rules so that we would synchronize again.
16. Did we have the same goal? At first the other seemed to be cooperative. Then I realised that he didn't, I said to myself that we didn't have the same objective. Sometimes we would get into the same rhythm and then it would stop. [...]. The other would do quite long sequences choosing the same colour, then he would change after 2 or 3 repetitions.

Specification of the Artificial agent algorithm

The artificial agent (AA) selected its target according to the probability for the player to choose a specific color after a given history. It stored the frequency that the participant chose each target after each possible history of four elements composed by two choices and two outcomes (**see table S1**). We call the probability of the player choosing the black card P_{black}. In Competitive trial blocks, the AA will choose the black card with probability $1 - P_{black}$, while in Cooperative trial blocks, it will choose the black card with probability P_{black}. A cooperative choice of the AA is defined as an AA choice following the most likely target chosen by the participant. Thus, even in competitive trial blocks, the AA can make a cooperative choice. Since the algorithm needs to be initialized, we arbitrarily defined the first five trials as random (the AA plays the black target with probability 0.5). The possible combinations that are not encountered during these initialization trials were assigned with a probability of choosing the black target of 0.5.

H1: $BWBW$	H2: $BWB\overline{W}$	H3: $BWRW$	H4: $BWR\overline{W}$
H5: $B\overline{W}BW$	H6: $B\overline{W}B\overline{W}$	H7: $B\overline{W}RW$	H8: $B\overline{W}R\overline{W}$
H9: $RWBW$	H10: $RWB\overline{W}$	H11: $RWRW$	H12: $RWR\overline{W}$
H13: $R\overline{W}BW$	H14: $R\overline{W}B\overline{W}$	H15: $R\overline{W}RW$	H16: $R\overline{W}R\overline{W}$

$$\mathrm{W} = \textbf{Win} \quad \mathrm{B} = \textbf{Black target chosen}$$
$$\overline{\mathrm{W}} = \textbf{Lose} \quad \mathrm{R} = \textbf{Red target chosen}$$

Table S1. Exhaustive (16) possible histories Hi of outcomes (Win/Lose) and choices (Black chosen/red chosen) used by the algorithm to track the probability that the participant plays black.

Description of computational models

The models described below are built to predict the probability to choose one specific target “*a*” or “*b*”. We define the probability to stay as $\begin{cases} p^{red} \; if\ previous\ choice\ was\ red \\ p^{black} \; if\ previous\ choice\ was\ black \end{cases}$ to match the terminology used in the behavioral analysis.

Reinforcement learning model

Reinforcement learning (RL) consisted of directly linking action or state and outcome to predict future rewards after performing a particular action or being in a particular state. In our experiment, we updated action value with the Rescola-Wagner rule:

$$V_{t+1}^{a} = V_{t}^{a} + \alpha * \delta_t \qquad \text{Eq 1}$$

$$\delta_t = R_t - V_t^{a} \qquad \text{Eq 2}$$

Where α is the learning rate. The reward prediction error δ_t is defined as the difference between the reward at trial *t*, R_t and the expected value of the choice *a* at trial *t*, V_t^{a}. Then the probability to choose action *a* is:

$$p^{a} = s(V^{a} - V^{b}) \qquad \text{Eq 3}$$

With $s(z) = {}^{1}/_{1 + e^{-\beta z}}$ the sigmoid function when β is a free parameter to capture the stochasticity of the participant’s behavior (i.e. the exploration/exploitation trade-off). We defined the probability to stay as $\begin{cases} p^{red} \; if\ previous\ choice\ was\ red \\ p^{black} \; if\ previous\ choice\ was\ black \end{cases}$. We used the same definition of the probability to stay for other models.

Fictitious play

In game theory, one can infer the probability that the other chooses one particular action and choose one’s own action to maximize one’s expected reward. This model is called a first order fictitious play model. Thus, the opponent’s probability p^{*} of choosing an action *a* is dynamically updated by tracking the choice history of the opponent:

$$p_{t+1}^{*} = p_t^{*} + \eta * \delta_t^{p} \qquad \text{Eq 4}$$

$$\delta_t^{p} = P_t - p_t^{*} \qquad \text{Eq 5}$$

Where η is the learning rate. The reward prediction error δ_t^{p} is defined as the difference between the expected action of the opponent at trial *t*, p_t^{*}, and the actual other’s choice on trial *t*, $(P_t = 1)$ if the other’s action is *a* and $(P_t = 0)$ if it is *b*. Then the probability to choose action *a* depends on the payoff matrix. In the competitive setting of our game we can derive the probability *q* that the participant chooses action $a = \text{“}Red\ card\text{”}$ using the sigmoid function, the payoff matrix, and the probability that the other chooses action $a = \text{“}Red\ card\text{”}$:

$p = s(2q^{*} - 1)$ Eq 6

p^{*} is the inferred probability that the other chooses $a = \text{“}Red\ card\text{”}$. Because the payoff matrix is the same for the participant in both Competitive and Cooperative trial blocks, the mode of interaction has no impact on the decision stage. However, considering the other’s decision rule, it would be different under competitive or cooperative assumption:

$$\begin{aligned} q &= s(2p^{*} - 1) \quad \text{in cooperative mode} \\ q &= s(1 - 2p^{*}) \quad \text{in competitive mode} \end{aligned} \qquad \text{Eq 7}$$

Then, the fictitious agent uses the inferred probability that the other chooses *a*, the payoff matrix and the other temperature to compute a decision value.

$$DV = \frac{p^{*}}{Temperature} * \big(Payoff(self = a, other = a) - Payoff(self = a, other = \bar{a})\big) + \frac{(1-p^{*})}{Temperature} * \big(Payoff(self = \bar{a}, other = a) - Payoff(self = \bar{a}, other = \bar{a})\big) \qquad \text{Eq 8}$$

Influence model

Another strategy could be to take into account how one's own actions influence the other's future actions. Thus, to compute the probability of updating of the other's strategy, we replaced update of opponent strategy (Eq. 4) in the player decision rule (Eq. 7). Then with a Taylor expansion taking η close to 0, we added the influence terms (Δp : influence update signal of the participant, Δq : influence update signal of the other):

$$\Delta q \approx +\eta 2\beta q_t(1-q_t)(P_t-p_t^*) \quad \text{Eq 9}$$

$$\Delta p \approx +\eta 2\beta p_t(1-p_t)(Q_t-Q_t^*) \quad \text{in cooperative}$$
$$\Delta p \approx -\eta 2\beta p_t(1-p_t)(Q_t-Q_t^*) \quad \text{in competitive} \quad \text{Eq 10}$$

Thus, in the competitive mode, there is only a sign difference between the term of influence of the two players which is not the case in the cooperative setting. A player can thus incorporate the influence of his/her action on the strategy of the other player:

$$p_{t+1}^* = p_t^* + \eta_1(P_t-p_t^*) + \eta_2 2\beta p_t^*(1-p_t^*)(Q_t-q_t^{**}) \quad \textit{in cooperative}$$
$$p_{t+1}^* = p_t^* + \eta_1(P_t-p_t^*) - \eta_2 2\beta p_t^*(1-p_t^*)(Q_t-q_t^{**}) \quad \textit{in competitive} \quad \text{Eq 11}$$

$$q_{t+1}^* = q_t^* + \eta_1(Q_t-q_t^*) + \eta_2 k_1 2\beta q_t^*(1-q_t^*)(P_t-p_t^{**}) \quad \text{Eq 12}$$

The Influence model update rules. p_t^* Is the predicted opponent strategy. P_t is the opponent choice and then $(P_t-p_t^*)$ is the action prediction error. The influence update is due to the $(Q_t-q_t^{**})$ term. Thus Q_t is the player's own action and q_t^{**} the inferred probabilities that the opponent has of the player themself (second-order beliefs). Thus, in the cooperative and competitive modes the influence will occur in the opposite directions. In the mixed-intention influence model, we decline p_{t+1}^* in $p_{t+1}^{coop\,*}$ and $p_{t+1}^{comp\,*}$.

To compute the p_t^{**} and q_t^{**}, we invert the decision function (Eq. 7):

$$q_t^{**} = \frac{1}{2} - \frac{1}{2\beta}\ln\left(\frac{1-p^*}{p^*}\right)$$
$$p_t^{**} = \frac{1}{2} + \frac{1}{2\beta}\ln\left(\frac{1-p^*}{p^*}\right) \quad \text{in competitive} \quad \text{Eq 13}$$
$$p_t^{**} = \frac{1}{2} - \frac{1}{2\beta}\ln\left(\frac{1-q^*}{q^*}\right) \quad \text{in cooperative}$$

As for the fictitious agent, the influence learner uses the inferred probability that the other chooses *a*, the payoff matrix and the other temperature to compute a decision value (Eq 8).

k-ToM model

The k-ToM model is defined as in [1]. An economic game under game theory is defined by a utility table $U(a^{self}, a^{other})$ to represent the payoff to players according to the actions of self,(a^{self}) and the other player (a^{other}). In our experiment, this utility table varies between Competitive and Cooperative blocks (**see Fig 1a**.). Because participants make a binary choice, a^{self} and a^{other} take the value of a=0 for one option and a=1 for the other option. According to Bayesian decision theory, agents try to maximize their expected value V = E[$U(a^{self}, a^{other})$]. We assume that agents use a softmax function as a decision rule:

$$P(a^{self}=1) = s\left(\frac{V^1-V^2}{\beta}\right) \quad \text{Eq 13}$$

$P(a^{self}=1)$ is the probability that the agent chooses option $a^{self}=1$. s is the sigmoid function and β is a free parameter called inverse temperature and controls for the magnitude of behavioral noise. The value of each action depends on the probability of other's action with $p^{other} = P(a^{other}=1)$ and the utility table $U(a^{self}, a^{other})$:

$$V^i = p^{other} * U(a^{self}=i, a^{other}=1) + (1 - p^{other}) * U(a^{self}=i, a^{other}=1) \quad \text{Eq 14}$$

One key hypothesis of this model is that we consider that the other agent is itself a k-ToM agent. It means that the other agent has the same decision policy as equation 13. Thus, while the agent tracks p^{other}, the other track p^{self} to construct a recursive reasoning. This recursion induces distinct levels of ToM sophistication between the two agents, impacting how agents update their subjective

prediction of p^{other}. [1] k-ToM agents are defined according to the way they update this prediction of p^{other} starting from 0-ToM. Definition of higher level of reasoning is based on the level 0, for which $P\left(a^{other}=1\right)=s(x_t^0)$, with the log-odd x_t^0 varying with a volatility σ^0. The updating rule for the hidden state x_t^0 follows the Bayes-optimal probabilistic scheme :

$$q(x_{t+1}^0) \propto p(a_{t+1}^0 \mid x_{t+1}^0) \int q(x_t^0) p(x_{t+1}^0 | x_t^0) dx_t^0 \qquad \text{Eq 15}$$

With $p(x_{t+1}^0 | x_t^0)$ the 0-ToM's prior belief on the volatility of the log-odd, and $q(x_t^0) \equiv p\left(x_t^0 | a_{1:t}^{other}\right)$, the posterior belief about the log-odds x_t^0 at trial t after the observation of all previous actions a^{other}. Thus, one can derive the 0-ToM's learning rule:

$$\hat{p}_{t+1}^{other} \approx s\left(\mu_t^0 \Big/ \sqrt{1 + {3(\Sigma_t^0 + \sigma^0)} \big/ {\pi^2}} \right) \qquad \text{Eq 16}$$

$$\mu_t^0 \approx \mu_{t-1}^0 + \Sigma_t^0 (a_t^{other} - s(\mu_{t-1}^0)) \qquad \text{Eq 17}$$

$$\Sigma_t^0 \approx \frac{1}{\frac{1}{\Sigma_{t-1}^0 + \sigma^0} + s(\mu_{t-1}^0)(1 - s(\mu_{t-1}^0))} \qquad \text{Eq 18}$$

Where μ_t^0 is the approximate mean of 0-ToM posterior distribution of $q(x^0)$ and Σ_t^0 is it's approximate variance. Thus μ_t^0 is the estimate of the 0-ToM log-odds at trial t and Σ_t^0 her subjective uncertainty about it.

A 1-ToM agent assumes that the other agent reasons with a 0 depth ToM. Thus, with the decision policy of a 0-ToM agent we can construct a 1-ToM agent. More specifically, in combining equation 13, 14 and 15, 1-ToM agent assumes that the probability for a 0-ToM agent to emit action $a^{other}=1$ is $p^{other} = s \circ v^1(x_t^1)$ (we use the symbol ∘ to refer to the composition of two functions defined as (g∘f)(x)=g(f(x))) with s the sigmoid function and v^1 the value for 0-ToM agent to choose option 1 :

$$v^1(x_t^1) = \frac{p_t^{self} * \Delta U_t^1 + \left(1 - p_t^{self}\right) * \Delta U_t^0}{\beta_t} \qquad \text{Eq 19}$$

With $\Delta \mathrm{U}_t^i = U\left(a^{self}=i, a^{other}=1\right) - U\left(a^{self}=i, a^{other}=0\right)$ which represents the incentive for the 1-ToM agent to choose option one if 1-ToM agent chooses option $a^{self}=i$. p_t^{self} for a 1-ToM agent is the same as p_t^{other} for 0-ToM agent, thus :

$$p_t^{self} \approx s\left(\mu_{t-1}^0 \Big/ \sqrt{1 + {3(\Sigma_{t-1}^0 + \sigma_t^0)} \big/ {\pi^2}} \right) \qquad \text{Eq 20}$$

To let the 1-ToM agent eventually learn how 0-ToM agent learns about herself, and act in consequence, the 1-ToM agent assumes that hidden states x_t^1 vary across trials with volatility σ^1, which leads to a meta learning rule similar to equation 16, 17, 18.

In a more general fashion, an agent of depth $k \geq 2$ considers that the other agent is a lower sophistication κ-ToM agent ($\kappa \leq \mathrm{k}$), but this sophistication has to be learned in addition to the hidden states x^κ that track the opponent's learning and decision making. Thus a k-ToM agent continuously tracks all possible other's sophistication levels and it's associate action probability $p^{other,\kappa} = s \circ v^\kappa(x^\kappa)$ that he will choose $a^{other}=1$.

$$p_t^{other} = \sum_{l<\kappa} \lambda_t^{k,\kappa} * p_t^{other,\kappa} \qquad \text{Eq 21}$$

$$p_t^{other} \approx s \circ \tilde{v}^\kappa\left(\mu_{t-1}^{k,\kappa}, \Sigma_{t-1}^{k,\kappa}\right) \qquad \text{Eq 22}$$

$$\lambda_t^{k,\kappa} \approx \left[\frac{\lambda_{t-1}^{k,\kappa} * p_t^{other,\kappa}}{\sum_{\kappa'<\kappa} \lambda_{t-1}^{k,\kappa'} * p_t^{other,\kappa'}}\right]^{a_t^{other}} \left[\frac{\lambda_{t-1}^{k,\kappa} * (1 - p_t^{other,\kappa})}{\sum_{\kappa'<\kappa} \lambda_{t-1}^{k,\kappa'} * (1 - p_t^{other,\kappa'})}\right]^{1 - a_t^{other}} \qquad \text{Eq 23}$$

$$\mu_t^{k,\kappa} \approx \mu_{t-1}^{k,\kappa} + \lambda_t^k \Sigma_t^{k,\kappa} W_{t-1}^\kappa \left(a_t^{other} - s \circ v^\kappa(\mu_{t-1}^{k,\kappa})\right) \qquad \text{Eq 24}$$

$$\Sigma_l^{k,\kappa} \approx \left[\left(\Sigma_{l-1}^{k,\kappa} + \sigma^k\right)^{-1} + s' \circ v^k\left(\mu_{t-1}^{k,\kappa}\right) \lambda_t^\kappa {W_{t-1}^\kappa}^T W_{t-1}^\kappa\right]^{-1} \qquad \text{Eq 25}$$

Where λ_t^k is k-ToM's probability that her opponent is κ-ToM, W^κ is the gradient of v^κ with respect to the hidden states x^κ. Here, v^k is obtained by the recursive injections of equation 5 in equation 1, as we have already done to obtain equation 4. $\tilde{v}^\kappa$ is defined in terms of the expectation operator:

$E\left[s \circ v^{\kappa}\left(\mu_{t-1}^{k,\kappa}, \Sigma_{t-1}^{k,\kappa}\right)\right] = s \circ \tilde{v}^{\kappa}\left(\mu_{t-1}^{k,\kappa}, \Sigma_{t-1}^{k,\kappa}\right)$. Equation 3 and 5 have been estimated using a Variational approach to approximate Bayesian inference.

Two Experts model

For models using the payoff matrix to update hidden states, making a difference between the Cooperative and Competitive modes (i.e. k-ToM and the Influence model), we fitted three models in different settings: competitive, cooperative or mixed intentions. When considering mixed-intentions models (k-TOM and influence models), we made the assumption that the cooperative expert and the competitive expert come from the same model (i.e. influence model or k-TOM) because, from the point of view of the participants, there is no indication that there are two modes of interaction. Therefore, it is more parsimonious to assume that a single process (i.e. same computational model for both experts) is engaged along the task.

For the mixed-intentions setting, we ran the competitive and cooperative models in parallel, avoiding the need for the payoff matrix to be learnt. On the first trial, each expert gives a prior probability that the other would choose the "*action a*" ($\mathrm{p}_{\mathrm{coop},0}^{*} = \mathrm{p}_{\mathrm{comp},0}^{*} = 0.5$), then each expert follows its own walk in generating on each trial the probability that the other will choose option a for each possible mode of interactions, $\mathrm{P}_{\mathrm{comp}}^{\mathrm{a}}$ and $\mathrm{P}_{\mathrm{coop}}^{\mathrm{a}}$. We then transformed the probability with the sigmoid function to have values ranging from $-\infty$ to $+\infty$. We have a binomial choice configuration thus $V^{a} = -V^{b}$ in both competitive and cooperative settings. Thus, as V_{i}^{a} and V_{i}^{b} get close to zero, uncertainty for *i*, the other's intention, increases. We defined the reliability of the intention *i* as the absolute value of V_{i}^{a} and the probability that the other intention is cooperative as the sigmoid function of the difference in reliability between the two modes:

$$P_{coop}^{t} = \frac{1}{1+e^{\beta\left(\left|V_{coop}^{t}\right|-\left|V_{comp}^{t}\right|-\delta\right)}} \qquad \text{Eq 26}$$

where β is the inverse temperature controlling for the stochasticity of the mode of interaction and δ is the bias towards cooperative mode. To motivate our definition of the reliability signal, we tested 4 definitions of reliability signals for the winning model:

- $|DV_{competitive}|$ - $|DV_{cooperative}|$ Difference of unsigned DV
- $|PE_{competitive}|$ - $|PE_{cooperative}|$ Difference of unsigned PE [2]
- $PE_{competitive}$ – $PE_{cooperative}$ Difference of PE [3]
- $Entropy_{competitive}$ – $Entropy_{cooperative}$ Difference of entropy: $-p * log(p) - (1-p) * log(1-p)$ [4]

When we performed a formal Bayesian model selection between these 4 reliability difference measures, the model with the 'Difference of unsigned DV' as a controller was the best model (protected exceedance probability pEP=0.83). Moreover, any mixed-intention influence model with one of these 4 reliability difference signals performs better than all the other 16 models presented in Fig 2.b. This demonstrates the robustness of the mixed-intention influence model, regardless of the chosen reliability difference measure.

Then, with $P_{comp}^{a,t}$ and $P_{coop}^{a,t}$ we computed the decision value given the Competitive and Cooperative payoff matrix, DV_{comp}^{a} and DV_{coop}^{a} respectively and weighted them by the probability of the corresponding mode of interaction to compute the total decision value:

$$DV^{t} = P_{coop}^{t} * DV_{coop}^{a} + \left(1 - P_{coop}^{t}\right) * DV_{comp}^{a} \qquad \text{Eq 27}$$

We call $P_{coop}^{t} * DV_{coop}^{a}$ the cooperative component of the model and $\left(1 - P_{coop}^{t}\right) * DV_{comp}^{a}$ the competitive component of the model. The sigmoid function s generated the probability of selecting choice *a* at trial t:

$$p^{a,t} = s(DV^{t}) \qquad \text{Eq 28}$$

Finally, the reward prediction error was defined as the reward at trial t for action a:

$$PE = R^{a,t} - p^{a,t} \qquad \text{Eq 29}$$

Active inference model

For this model based on [5], we adopted the partially observable Markov decision process (POMDP) framework which is a way of describing transitions among states under the hypothesis that the

probability of the next state depends only on the current state. The partially observed aspect of the Markovian process means that states are not directly observable and have to be inferred though a set of (noisy) observations.

Active inferences are composed of a tuple (P, Q, R, S, A, U , Ω) :

- Ω is a finite set of possible observations
- A is a finite set of possible action
- S is a finite set of hidden states
- U is a finite set of control states
- R is the *generative process* over observation $\tilde{o} \in \Omega$, hidden states $\tilde{s} \in S$, and action $\tilde{a} \in A$

$$R(\tilde{o}, \tilde{s}, \tilde{a}) = Pr(\{o_0, \dots, o_t\} = \tilde{o}, \{s_0, \dots, s_t\} = \tilde{s}, \{a_0, \dots, a_{t-1}\} = \tilde{a})$$

- P is the *generative model* over observation $\tilde{o} \in \Omega$, hidden states $\tilde{s} \in S$, and control states $\tilde{u} \in U$
  $P(\tilde{o}, \tilde{s}, \tilde{u}|m) = Pr(\{o_0, \dots, o_T\} = \tilde{o}, \{s_0, \dots, s_T\} = \tilde{s}, \{u_0, \dots, u_T\} = \tilde{u})$ with parameters θ.
- Q is the *approximate posterior* over hidden and control states
  $Q(\tilde{s}, \tilde{u}) = Pr(\{s_0, \dots, s_T\} = \tilde{s}, \{u_0, \dots, u_T\} = \tilde{u})$ with parameters or expectation $(\hat{s}, \hat{\pi})$, where $\pi \in \{1, \dots, K\}$ is a policy that indexes a sequence of control states
  $(\tilde{u}|\pi) = (u_t, \dots, u_T|\pi)$

Firstly, *generative process* describes the transition probabilities among hidden states which generate observations. Transition probabilities depend on actions which are sampled from *approximate posterior* belief about control states. Belief is formed using the *generative model* (denoted by *m*) of how observations are generated by hidden states. The G*enerative model* encodes belief and hidden states of the agent in term of expectation.

The active inference model assumes that both action and expectation minimize the free energy of observations. That is, expectation minimizes free energy and expectation of control states prescribes actions in each trial.

$$(\hat{s}, \hat{\pi}) = argmin\, F(\tilde{o}, \hat{s}, \hat{\pi}) \qquad \text{Eq 30}$$

$$\Pr(a_t = u_t) = Q(u_t|\hat{\pi}^*) \qquad \text{Eq 31}$$

With:

$$\begin{aligned} F(\hat{o}, \hat{s}, \hat{\pi}) &= E_Q[-\ln P(\tilde{o}, \tilde{s}, \tilde{u}|m)] - H[Q(\tilde{s}, \tilde{u})] \\ &= -\ln P(\tilde{o}\,|m) + D_{KL}\big[Q(\tilde{s}, \tilde{u}) \,||\, P(\tilde{s}, \tilde{u}|\tilde{o})\big] \end{aligned} \qquad \text{Eq 32}$$

The generative mode could be defined as three marginal distributions:

$$P(\tilde{o}, \tilde{s}, \tilde{u}|m) = P(\tilde{o}|\tilde{s})\, P(\tilde{s}|\tilde{u})\, P(\tilde{u}|m) \qquad \text{Eq 33}$$

Thus, heuristically, the decision consists firstly of figuring out which state is the most likely by optimizing its expectation according to free energy and the generative model. Then, after optimizing its posterior beliefs, an action is sampled from the posterior probability distribution over the control state. The environment generates a new observation given the selected action using the generative process and a new decision cycle begins.

For our experimental design, we have 8 possible observations:

Ω = { "Previous target *a,* loose"; "Previous target *a,* win"; "Previous target *b,* loose"; "Previous target *b,* win"; "Current target *a,* win"; "Current target *a,* loose"; "Current target *b,* win"; "Current target *b,* lose"}

We defined 20 hidden states:

S = {"Previous choice" x "previous reward" x "current correct answer" x "current mode of interaction" ; "Current target *a,* win"; "Current target *a,* loose"; "Current target *b,* win"; "Current target *b,* lose"}

The finite set of action is A = {"Choose target *b* "; "Choose target *a*"} bringing the agent from the 16 first possible hidden states to their corresponding 4 last hidden states which are {"Current target *a,* win"; "Current target *a,* loose"; "Current target *b,* win"; "Current target *b,* lose"}.

Log prior preferences over the observed states are C = [0 ; 1 ; 0 ; 1 ; 2 ; -1 ; 2 ; -1] meaning that the agent prefers to observe, in decreasing order, a current winning, then a previous winning, a previous defeat and finally a current defeat.

For each trial, prior beliefs about hidden states are equally spread between the four hidden states composed by {“Previous choice” x “previous reward”} leaving unknown the “current mode of interaction” and the “current good answer”.

To allow the agent to learn about the hidden state “current mode of interaction” we added concentration parameters about observation. Concentration parameters are prior about what hidden states lead to what observations, and can be viewed as the number of hidden states’ occurrences encountered in the past. We arbitrarily set this number to 2 for being in “cooperative” mode, when observing a previous winning, and 1 for being in the hidden state “competitive”. Inversely for a previous defeat, we set this parameter at 1 for being in the “cooperative” hidden state and 2 for being in the “competitive” state.

Hierarchical Gaussian Filter

The Hierarchical Gaussian Filter was constructed to model the agent's learning under a volatile environment [6,7]. We are interested in a binary state x_1^t of the environment at time *t* (for convenience we will often omit the time index *k*) :

$x_1^k \in \{$"$\mathbf{Card}\ a$ **is the good answer**" ; "**Card** b **is the good answer**" $\}$ is causing sensory input $\boldsymbol{u}$. Thus, we assume the following form of likelihood function:

$$p(u|x_1) = (u)^{x_1}(1-u)^{1-x_1} \quad \text{Eq 34}$$

Because x_1 is binary, it could be described by a single real number, x_2, the state at the next level of hierarchy. We then define the conditional prior density to map x_2 to the probability x_1 as a Bernnouilli law of parameter $s(x_2)$:

$$p(x_1|x_2) = s(x_2)^{x_1}\left(1-s(x_2)\right)^{1-x_1} \quad \text{Eq 35}$$

Where $s(x) = \frac{1}{1+e^{-x}}$ is the sigmoid function. This prior density gives us $x_1 = 1$ and $x_1 = 0$ equally probable for $x_2 = 0$ and for $x_2 \to +\infty$ or $-\infty$ we have respectively $x_1 \to 1$ or 0. Thus, x_2 is an unbounded parameter of the probability that $x_1 = 1$. In our example, a higher x_2 corresponds to a strong tendency for the red target to be the good answer. The only hypothesis on x_2 is that it evolves with time as a Gaussian random walk.

$$p\left(x_2^{(k)}|x_2^{(k-1)}, x_3^{(t)}\right) = N\left(x_2^{(k)}; x_2^{(k-1)}, \exp\left(\kappa x_3^{(k)} + \omega\right)\right) \quad \text{Eq 36}$$

Where ω and κ are two free parameters corresponding to the dispersion of the random walk. $x_3^{(k)}$ represents the log-volatility of the environment, meaning the tendency of the red target to be the good answer, and follows a Gaussian random walk. ω represents the volatility independent of the state x_3. Then we can apply the same approach to x_3 as we do to x_2, and so forth, to add as many levels as desired. Here we stop at the fourth level introducing a new free parameter representing the volatility of x_3 :

$$p\left(x_3^{(k)}|x_3^{(k-1)}, \vartheta\right) = N\left(x_3^{(k)}; x_3^{(k-1)}, \vartheta\right) \quad \text{Eq 37}$$

Then, using the Variational inversion method explained in [6], we can inverse the model to update hidden states given a regular sensory entry $u^{(k)}$. The approximation of the inversion assumes Gaussian posteriors at all levels, with means μ_i and precision (inverse of variance) π_i :

$$x_i^{(k)} \mid u^{(1)}, \ldots, u^{(k)}, \chi \sim N\left(\mu_i^{(k)}, \left(\pi_i^{(k)}\right)^{-1}\right) \quad \text{Eq 38}$$

with χ the set of all free parameters. Thus parameters μ_i and π_i are the sufficient statistic, to be updated after each input *u* as follows:

$$\mu_i^{(k)} = \hat{\mu}_i^{(k)} + \frac{1}{2}\kappa_{i-1} v_{i-1}^{(k)} \frac{\hat{\pi}_{i-1}^{(k)}}{\pi_i^{(k)}} \delta_{i-1}^{(k)} \quad \text{Eq 39}$$

$$\pi_i^{(k)} = \hat{\pi}_i^{(k)} + \frac{1}{2}\left(\kappa_{i-1} v_{i-1}^{(k)} \hat{\pi}_{i-1}^{(k)}\right)^2 \left(1 + \left(1 - \frac{1}{v_{i-1}^{(k)} \pi_{i-1}^{(k-1)}}\right)\delta_{i-1}^{(k)}\right) \quad \text{Eq 40}$$

With

$$v_i^{(k)} = \begin{cases} t^{(k)} \exp\left(\kappa_i \mu_{i+1}^{(k-1)} + \omega_i\right), & i = 1, \ldots, n-1 \\ t^{(k)}, & i = n \end{cases} \quad \text{Eq 41}$$

$$\hat{\mu}_i^{(k)} = \mu_i^{(k-1)} \ \text{by definition}$$

$$\hat{\pi}_i^{(k)} = \frac{1}{\sigma_i^{(k-1)} + v_i^{(k)}} \ \text{by definition}$$

$$\delta_i^{(k)} = \frac{\sigma_i^{(k)} + \left(\mu_i^{(k)} - \hat{\mu}_i^{(k)}\right)^2}{\sigma_i^{(k-1)} + v_i^{(k)}} \text{by definition}$$

Bayesian sequence learner

The n-BSL (Bayesian sequence learner) is a model which tracks probabilities of a certain outcome "a" given the previous *n* outcomes as a Gaussian function:

$$P(a = \text{"Red target is the good answer"} \mid S_i) = N(\mu_i, \sigma_i)$$

With S_i the sequence of the *n* last outcomes “a”. For each observation at time t, the update is a Laplace-Kalman rule:

$$\sigma_i^{t+1} = \frac{1}{\frac{1}{\sigma_i^t+\Omega}+s(\mu_i^t)*\left(1-s(\mu_i^t)\right)} \quad \text{Eq 42}$$

$$\mu_i^{t+1} = \mu_i^t + \left(\sigma_i^{t+1} + \Omega\right) * \left(a^t - s\left(\mu_i^t\right)\right) \quad \text{Eq 43}$$

With $a^t = 1$ if "Red target is the good answer" and $a^t = 0$ if "Target *a* is the good answer", $s(x) = \frac{1}{1+e^{-x}}$, and Ω is a free parameter representing prior volatility.

Win-Stay / Lose-Switch model

This model reproduces a heuristic behavior, precisely “I keep the same option if I just won, I switch if I just lost”. To implement that, we use two pseudo Q-values, $V^{stay} = 1$ for the action of stay and $V^{switch} = -1$ for the action of switch. Then we use the sigmoid function to compute the probability of choosing the same option as the previous trial:

$$p^{stay} = s\left(V^{stay} - V^{switch}\right) \quad \text{Eq 44}$$

Supplementary Note 1: additional behavioral data analyses

Mixed-intention influence model: complementary analyses

To give the reader an idea of the absolute fit of the model, we computed the balanced accuracy, which is a robust measure of fit for binomial prediction[8]. The balanced accuracy is ranged from 40% to 83% with a mean equal to 62%.

To determine whether the mixed-intention influence model could recover the observed effect of other's Cooperativity signature (interaction between the participant's outcome and the following choice by the AA to switch or not) on the probability to stay on the same target, we simulated 320 sets using the influence model from each of the 3 versions (i.e. the competitive expert alone, the cooperative expert alone and the mixed intentions version arbitrating between the cooperative and competitive experts) playing against the choice sequences that the artificial agent generated against real participants. In this approach the mixed-intention influence model adapts to the choices of the AA, but the choices of the AA **do not** depend upon the choices of the mixed-intention influence model. We randomly drew free parameters from a normal distribution centered at the mean of the observed parameters of participants and with the standard deviation observed in the data. The results of these logistic regressions are shown in Supplementary Fig 6. All three models are able to reproduce the win/stay-lose/switch strategy, <u>but</u> only the mixed-intention influence model is able to reproduce the subtle effect of the cooperativity signature of other on the strategy to stay. Moreover, the mixed-intention influence model tends to generate less switch after a switch in previous trial than the other experts alone. This confirms the validity and specificity of the winning model (for behavior generated by the cooperative expert alone or competitive expert alone, see **Supplementary Fig. 6 a,b**).

While our results suggest that the mixed-intention influence model best predicts and reproduces behavior of participants. It is possible that the tracking was not dynamic across trials but fixed throughout the entire experiment (for example with the same ratio of Competitive/Cooperative numbers of trial). To test this hypothesis, of a fixed arbitrator deciding between the two experts, we also tested a model using a static free parameter arbitrating between the two experts. The results of the Bayesian model selection still assigned the mixed-intention influence model to most participants, even after addition of this free parameter, which demonstrate the importance of the dynamic aspect of the tracking. Finally, because only the second order term of the influence model differentiates the two modes of interactions, we tested the contribution of the mentalizing term to the fit by removing this parameter. This led to a decrease of 5.39% (95% confident interval [2.47;-8.31]) in the log-likelihood, indicating the importance of the mentalizing term.

To check if our estimated parameters are identifiable, we generated 1000 datasets with the mixed-intention influence model playing against the choice sequences that the artificial agent generated. In this approach the mixed-intention influence model adapts to the choices of the AA, but the choices of the AA do not depend upon the choices of the mixed-intention influence model. We randomly drew free parameters from a normal distribution centered at the mean of the observed parameters of participants and with the standard deviation observed in the data. We then estimated the free parameters recovered when fitting the mixed-intention influence model on these new datasets. We then made linear regressions on each generative parameter with all recovered parameters as regressors to see if the variance of a specific generative free parameter is best explained by its corresponding recovered free parameter. A score of 1 mean that the variance of the generative parameter is totally explained by the variance of the recovered parameter. **Supplementary fig 4** indicates that every generative free parameter is best explained by its corresponding recovered free parameter. Moreover, as can be seen in Supplementary Table 1, there are covariations between the free parameters of our generative model (mixed-intention influence learning). However, we do not interpret the value of these parameters. In addition, we wanted to keep the mixed-intention influence model exactly the same as that used in the literature to relate and compare our results to it. Obviously, it would have been possible to twist these models to avoid such correlations between parameters, but this would have been at the expense of the difficulty of making comparisons with the existing models.

To verify that our models make distinct prediction on behavior, we generated 5 datasets for each of our 31 participants independently (total of 155 datasets). To do so we used randomly generated free parameter with mean value the fitted free parameters of each participant. Generative models made decision facing a non-contingent Artificial Agent (AA, i.e. we used the real sequence of choices that

participant observed during the experiment). We found that no model produce behavior that could be confounded with the winning model (Mixed-intention influence model). Moreover, the behavior generated by the mixed-intention influence model could not be recover by another model in our model set, see confusion matrix in **Supplementary figure 5.**

Evidence for separate cooperative and competitive experts

Below, we demonstrate the distinguishability and independence of the two experts at the computational and behavioral levels.

First, it should be noted that the predictions of the cooperative expert are not always completely anti-correlated to the predictions of the competitive expert. Indeed, the model was designed in a way that allows the two experts be either correlated, anti-correlated or not correlated at all. The two experts start with the same prediction on the choice of the participant ($p^*_{coop,0} = p^*_{comp,0} = 0.5$), but then have an independent evolution across trials (see Equations 11). Thus, we can distinguish 3 main cases. First, if the first order prediction error $\eta_1(P_t - p^*_{coop,t})$ is globally high with respect to the second order prediction error $\eta_2 2\beta p^*_{coop,t}(1 - p^*_{coop,t})(Q_t - q^{**}_{coop,t})$, strategies of both experts will be correlated all along the game (because the first order PE is identical for both experts). Second, if the two PE (i.e first and second order) are of the same order of magnitude, the cooperative and competitive strategies will not be correlated at all. Third, if the second order PE is globally high with respect to the first order PE, the two strategies will be anti-correlated. These 3 cases demonstrate the validity of the 'mixture of experts' framework for modeling our task since the 2 experts are not structurally anti-correlated. Thus, our model cannot be reduced to a single expert that either cooperates or competes.

Second, **Supplementary Figure 1** displays the correlations between the predictions of the 2 experts for each of the 31 participants. They show either a correlation, no correlation or anti-correlation between the predictions of the 2 experts. Thus, the two separate experts can be distinguished and they are not anti-correlated. Choice probabilities of the two experts were significantly correlated at the group level: R^2=0.21 (CI[0.10;0.36]) (p<0.0001). However, this correlation is not forced by the equations of the models (i.e. this correlation is conjectural and is NOT structural) since it depends upon free parameters, and the interaction between the choices of the participants and the choices of the AA.

Third, we observed no correlation between the reliability of the cooperative expert and the reliability of the competitive expert (R^2=0.005, p=0.148) (**Supplementary figure 2**). This indicates that the two strategies (cooperative and competitive) are not reliable at the same time. This also demonstrates the importance of the second order prediction error term to differentiate the two strategies.

Fourth, the cooperative and competitive components of the decision value are correlated with only 0.4% of common variance (R^2=0.0044, p= 0.001) (**Supplementary figure 3**). Note that when the decision value computed by the cooperative expert (respectively competitive expert) is around 0, the variance of the decision value of the competitive expert (respectively cooperative expert) is large. This indicates that when one expert is reliable, the other one is often unreliable. Globally, when one expert has a precise prediction regarding the best future choice for the participant, the other expert often proposes a less reliable choice. Thus, overall, the two experts are complementary.

Supplementary Note 2: additional fMRI data analyses

Neural correlates of higher order feature of the interaction

We searched for brain regions computing the decision value for staying on the same target for the mixed-intention influence model and for classic reinforcement learning to compare them for higher order inferences. To do this, we ran two GLMs (GLM5 and GLM6) containing the decision value for staying on the same target as the only parametric regressor at the time of choice. In GLM5, the decision value for staying was computed with the mixed-intention influence model whereas in GLM6 it was computed with a reinforcement learning model. We found that the ventral striatum coded the decision value for staying on the same target positively, as computed by the mixed-intention influence model (x,y,z = 14, 11,-2; $p < 0.05$ whole-brain FWE corrected at the cluster level, initial cluster forming threshold of $p<0.001$, see **Supplementary Fig. 9a**). Comparison of the neural correlates of the decision value of the two models with a paired t-test revealed that Ventral Striatum (x,y,z=6,12,0), bilateral dlPFC (x,y,z=-36, 33, 44 and x,y,z= 30,24,42) and MTG (x,y,z=65,-56,-8, $p<0.05$ FWE corrected, initial cluster forming threshold of $p<0.001$) were encoding higher order features of the task (see **Supplementary Fig. 9b**).

Neural activity is specific to the attribution of intention, but not the real intention

It is possible that the observed DLPFC/IPS region activations are only due to the difference in behavior of the artificial agent between the Competitive and Cooperative blocks. To test this hypothesis, we conducted another GLM (GLM3), separating trials according to the Cooperative blocks vs Competitive blocks (and not according to classification of trials by the controller). We observed no difference in brain activation, even at a lower threshold ($p>0.01$), indicating that the PE difference observed between the trials classified as competitive by the controller and the trials classified as cooperative were due to the effective tracking of attribution of intentions, and not to PE differences between the two (unsignaled) types of trial blocks. Direct paired t-test comparison between PE for trials classified as competitive > cooperative (i.e. $(\Delta>0)>(\Delta<0)$) and PE from the comparison between blocks of Competitive and Cooperative trials showed the engagement of the same regions (right angular gyrus x,y,z=48,-50,32, right dlPFC x,y,z= 30,8,53 and left angular gyrus x,y,z=-33,-59,44 ($p<0.05$ few, initial cluster forming threshold of $p<0.001$). The left dlPFC was also engaged at a lower threshold (x,y,z=-39,-2,26; $p<0.01$ FWE, initial cluster forming threshold of $p<0.005$).

The activities we observed could be due to more volatility in the rewarded target, therefore we ran a fourth GLM (GLM4) to control for the volatility of the Artificial Agent. Indeed, the probability that the computer switches target was 11% higher in Competitive than in Cooperative trials ($p<0.0001$, 95%CI [8.8%; 14%]). However, when adding the trial by trial probability, that the artificial agent switches target as a non-orthogonalized regressor, the right dlPFC (x,y,z=30,9,42) and angular gyrus (x,y,z=51,-50,33) were still more positively correlated with PE in trials classified as competitive by the controller compared to those classified as cooperative. This result indicates that those activations are not due to the volatility of the competitive condition.

Supplementary data

Supplementary tables

Supplementary Table 1. Mean of correlation coefficient between free parameter across all participant. First line is the mean value of the correlation. Second line is the p-value corrected with Bonferroni for multiple correlation, and confidence interval with a confident level of 99.76% related to figure 2b.

FREE PARAMETER	WEIGHT OF FIRST ORDER PE	WEIGHT OF SECOND ORDER PE	OPPONENT'S TEMPERATURE	CONTROLLER SLOPE (β)	CONTROLLER BIAIS (δ)	COMPETITIVE TEMPERATURE	COOPERATIVE TEMPERATURE
**WEIGHT OF FIRST ORDER PE**	1						
**WEIGHT OF SECOND ORDER PE**	0.026	1					
	1<br>[-0.038; 0.090]						
**OPPONENT'S TEMPERATURE**	0.270	0.059	1				
	0<br>[0.173; 0.367]	1<br>[-.046; 0.164]					
**CONTROLLER SLOPE (β)**	-0.079	0.099	-0.124	1			
	0.004<br>[-0.140; -0.018]	0.057<br>[-0.002; 0.199]	0<br>[-0.197; -0.051]				
**CONTROLLER BIAIS (δ)**	0.275	0.077	-0.177	0.139	1		
	0<br>[0.174; 0.376]	0.126<br>[-0.009; 0.164]	0.002<br>[-0.310; -0.045]	0.002<br>[0.040; 0.238]			
**COMPETITIVE TEMPERATURE**	0.203	-0.201	-0.149	-0.020	-0.024	1	
	0<br>[0.086; 0.321]	0.002<br>[-0.346;-0.057]	0.015<br>[-0.280; -0.018]	1<br>[-0.077; 0.036]	1<br>[-0.122; 0.073]		
**COOPERATIVE TEMPERATURE**	-0.414	-0.061	-0.009	-0.027	0.000	0.021	1
	0<br>[-0.557;-0.272]	0.004<br>[-0.110; -0.012]	1<br>[-0.033; 0.016]	0.046<br>[0.000; 0.054]	1<br>[-0.050; 0.051]	1<br>[-0.024; 0.065]	

Supplementary results table of GLM1, related to Figure 4c.

Table 2. Brain regions that covary with difference in reliability of interaction mode

	MNI peak cluster coordinates				
	x	y	z	k	Z score
**Difference in reliability (Δ=Rcoop -Rcomp)**					
Ventral Striatum	14	12	-2	4506	5.08
Left hippocampus	-32	-44	-2	1427	5.18
mPFC	6	46	-6	721	4.46
Right hippocampus	32	-36	10	6735	4.48
Left superior frontal gyrus	-15	-10	69	2528	4.01
**Rcomp – Rcoop**					
No brain region					

** cluster reported at $p<0.05$ FWE whole brain cluster corrected (initial cluster-forming threshold of $p<0.001$ uncorrected)

Supplementary results table of GLM2, related to Figure 5a.

Table 3. Brain regions encoding both competitive and cooperative prediction error.

	MNI peak cluster coordinates				
	x	y	z	k	Z score
**Positively**					
Right Dorsal Striatum	17	6	-12	21895	8.79
Left Dorsal Striatum	-14	3	-11	3488	8.78
Left Cerebellum	-44	-74	-45	6381	5.49
Right Cerebellum	29	-71	-29	5299	5.33
PCC	2	-35	38	1285	5.3
Right Angular/TPJ	45	-30	47	3581	4.71
Left Angular/TPJ	-54	-62	39	2044	4.34
**Negatively**					
No brain region					

** cluster reported at $p<0.05$ FWE whole brain cluster corrected (initial cluster-forming threshold of $p<0.001$ uncorrected)

Supplementary results table of GLM2, related to Figure 5c.

Table 4. Brain regions that responded differently for the reward prediction error of a trial estimated to be competitive rather than cooperative

	MNI peak cluster coordinates				
	x	y	z	k	Z score
**competitive > cooperative**					
Right dlPFC	35	11	36	1732	4.41
Right Angular/TPJ	50	-50	32	1758	4.32
Medial superior frontal gyrus	-2	30	47	505	3.82
**cooperative > competitive**					
No brain region					

** cluster reported at p<0.05 FWE whole brain cluster corrected (initial cluster-forming threshold of p<0.001 uncorrected)

Supplementary results table of connectivity analysis, related to Figure 6.

Table 5. Brain regions functionally connected with regions encoding the controller at the decision time.

**gPPI Analyses**	Seed	Peak regions	k	p-val FWE	Z score
**competitive > cooperative**	*mPFC + striatum*	-48 -44 58	363	<0.001	4.52
	mPFC + striatum	-2 -82 -36	153	0.037	4.34
	mPFC + striatum	38 34 34	150	0.040	4.33
	mPFC + striatum	-38 -78 -26	181	0.017	4.27
**cooperative > competitive**	mPFC + striatum	4 -46 16	744	<0.001	4.48

** cluster reported at p<0.05 FWE whole brain cluster corrected (initial cluster-forming threshold of p<0.001 uncorrected)

Supplementary figures

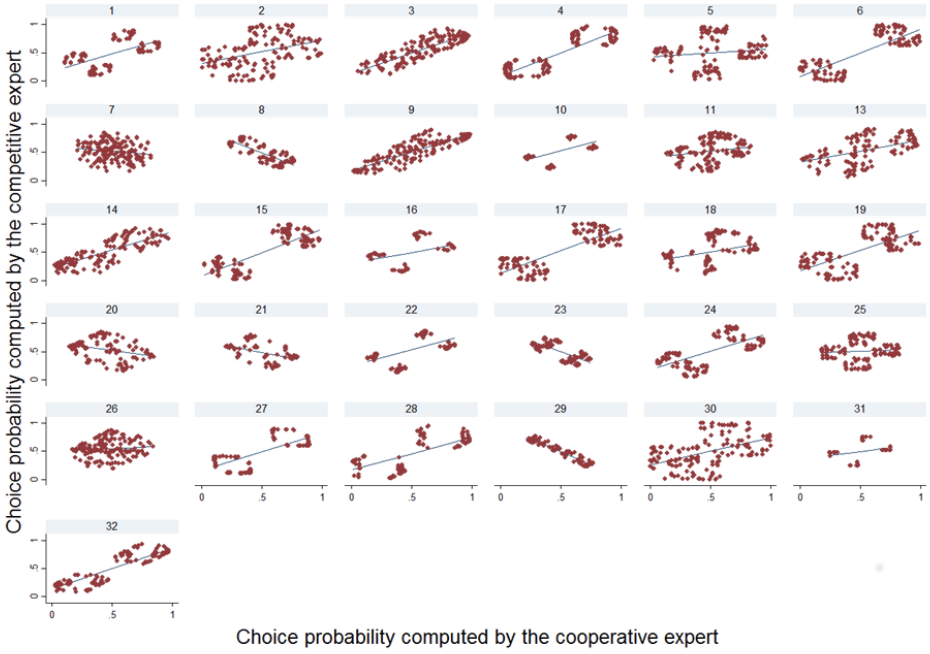

Supplementary figure 1. Correlations between choice probability of the two experts. Related to Computational models tracking intentions of the other agent in the Results section. Participant by participant correlations between choice probability computed by the cooperative expert (x-axis) and the competitive expert (y-axis). Each dot represents one trial. The line represents the linear regression.

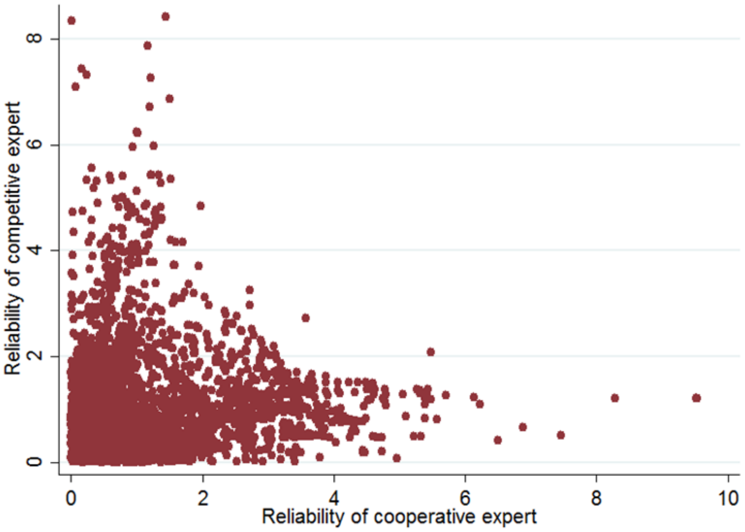

Supplementary figure 2. Relation between the two reliability of the two experts. Related to Computational models tracking intentions of the other agent in the Results section. Graph representing the reliability of the competitive expert according to the reliability of the cooperative expert. Each dot represents one trial. No correlation between the two reliability signals was found R^2=0.005 (p=0.148).

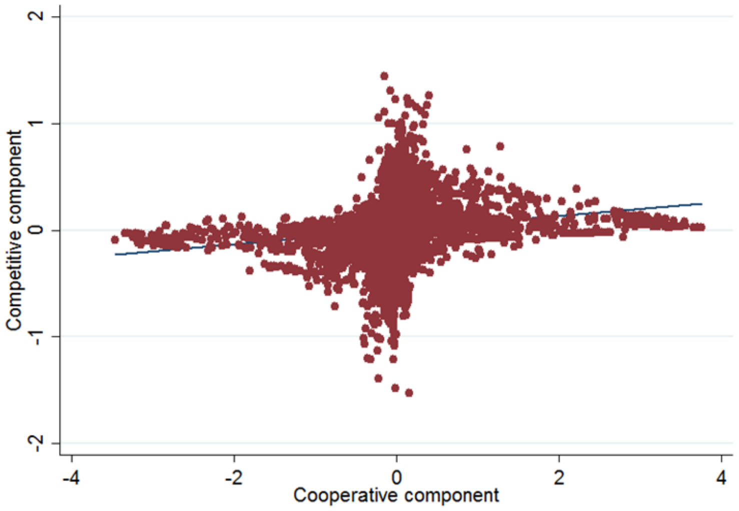

Supplementary figure 3. Relation between cooperation part of the Mixed-intentions Influence model and competition part. Related to Computational models tracking intentions of the other agent in the Results section. Graph representing the competitive component of the global decision value as a function of the cooperative component of the Mixed-intentions influence model. The global decision value is computed as follows: $DV = \mathrm{P(be\ in\ competitive)} * \mathrm{DV}_{\mathrm{competitive}} + \mathrm{P(be\ in\ cooperative)} * \mathrm{DV}_{\mathrm{cooperative}}$. The first term of the sum is called the competitive component; the second term of the sum is called the cooperative component. Each dot represents one trial. The line represents the linear regression. We found a slight correlation with only 0.4% of common variance (R^2=0.0045, p= 0.001).

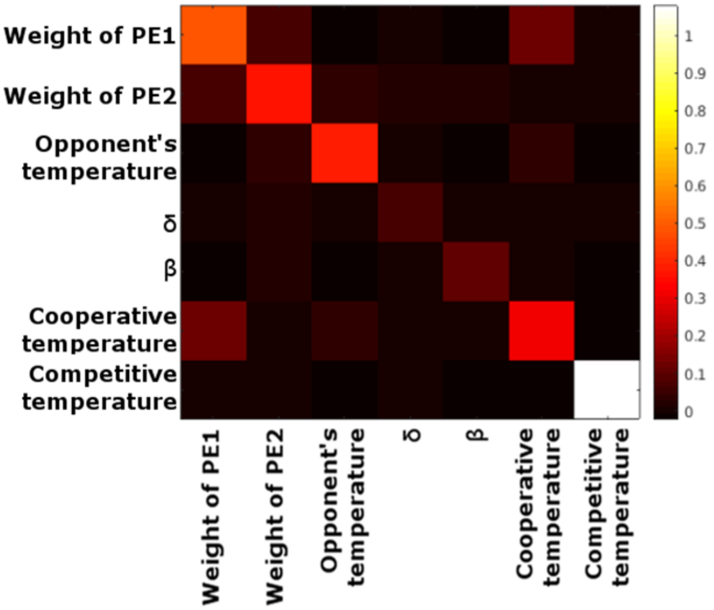

Supplementary figure 4. Parameter recovery matrix (full parameter set). Related to Computational models tracking intentions of the other agent in the Results section. Each line of the matrix shows the squared partial correlation coefficient between a given generated parameter and every recovered parameters (across 1000 simulations) (correlation values are color coded according to the color scale on the right side). Note that perfect recovery would exhibit a diagonal structure, where variations in each estimated parameter is only due to variations in the corresponding simulated parameter. Diagonal elements of the recovery matrix measure “correct estimation variability”, i.e., variations in the estimated parameters that are due to variations in the corresponding simulated parameter. In contrast, non-diagonal elements of the recovery matrix measure “incorrect estimation variability”, i.e., variations in the estimated parameters that are due to variations in other parameters. Strong non-diagonal elements in recovery matrices thus signal pairwise non-identifiability issues. Parameter 1 is the weight of first order PE (PE1), parameter 2 is the weight of second order PE (PE2), parameter 3 is the opponent’s temperature, parameter 4 is the slope of controller’s sigmoid (controller slope), parameter 5 is the center of controller’s sigmoid (controller’s bias), parameter 6 is the temperature of the cooperative expert, parameter 7 is the temperature of the competitive expert.

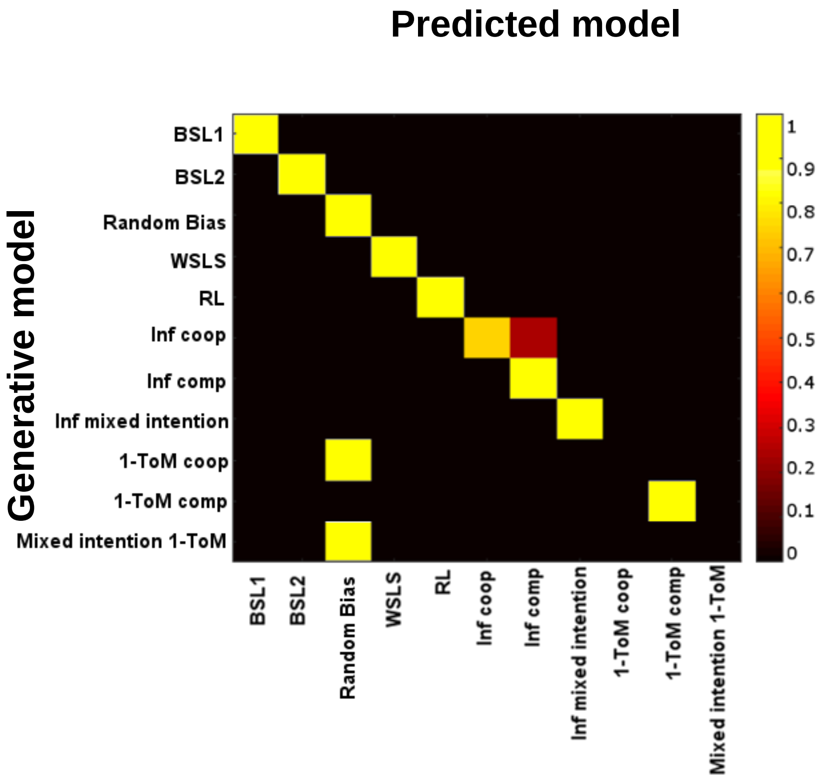

Supplementary figure 5. Confusion matrix. Related to Computational models tracking intentions of the other agent in the Results section. We generated data with free parameters randomly selected from a normal distribution centered on the mean of participants' free parameters, and with variance equal to variance of these free parameters. Using this procedure, 5 datasets for each of our 31 participants were generated (total of 155 datasets). Then, we ran a model selection on these generated datasets, and we repeated this procedure for each model. The rows are the generative models. The columns are the predicted model. The colors represent the probability that one model is more frequent (in our population of generated dataset) than another given a Bayesian model selection (Exceedance probability).

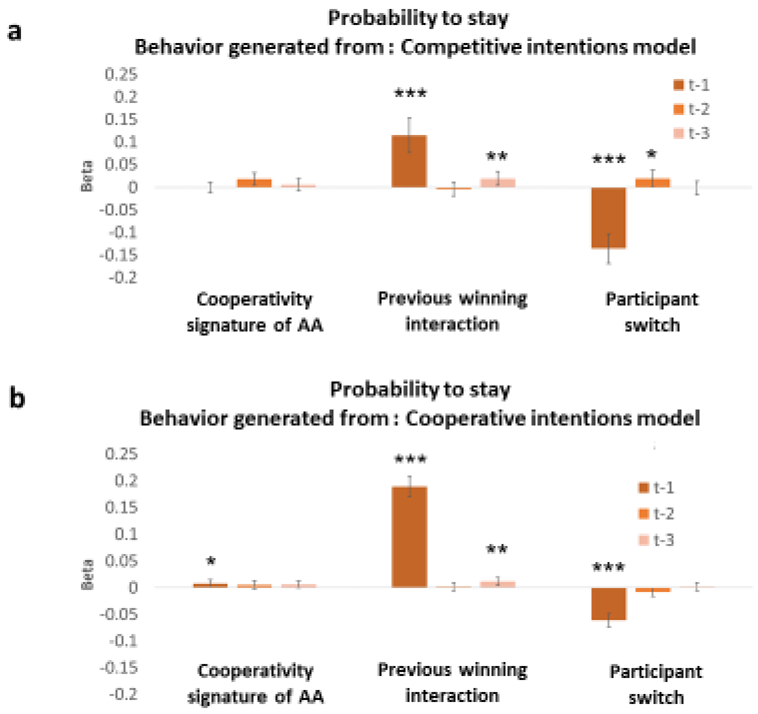

Supplementary figure 6. Model-based generative analysis. Related to Computational models tracking intentions of the other agent in the Results section. We generated n=320 independent sets of data using free parameters from a normal distribution with mean and standard deviation calculated from the "Influence models" in competitive (a) and cooperative (b) modes, fitted to the population. We generated these data sets against the fixed sequences of choices that the artificial agent made against participants during the experiment. We regressed the interaction of the previous outcome and action of the artificial agent (I win – AA stay / I lose – AA switch), the behavioral decision to stay after selecting a specific target at the previous trial based on the success or failure of the previous trial (Win) and the action to switch or stay of the artificial agent (Switch) in previous trials up to three trials back. Bars represent the marginal effect in percentage of each explicative variables on the probability to stay and the error bars are the 95% confidence interval. *p < 0.05, **p < 0.01, ***p < 0.001 (random-effect logistic regression, one-sided χ^2, not corrected for multiple comparison). Concerning the Competitive model **(a)** and the cooperativity signature of the AA at t-1,t-2 and t-3, the p-values are respectively p=0.968, p=0.360 and p=0.337. For the previous winning interaction at t-1,t-2 and t-3, the p-values are respectively p<0.001, p=0.822 and p=0.011 and finally, for the previous participant switch at t-1, t-2 and t-3, the p-value are p<0.001, p=0.044 and p=0.743. Concerning the Cooperative model **(b)** model and the cooperativity signature of the AA at t-1,t-2 and t-3, the p-values are respectively p=0.968, p=0.360 and p=0.337. For the previous winning interaction at t-1,t-2 and t-3, the p-values are respectively p<0.001, p=0.822 and p=0.011 and finally, for the previous participant switch at t-1, t-2 and t-3, the p-value are p<0.001, p=0.044 and p=0.743.

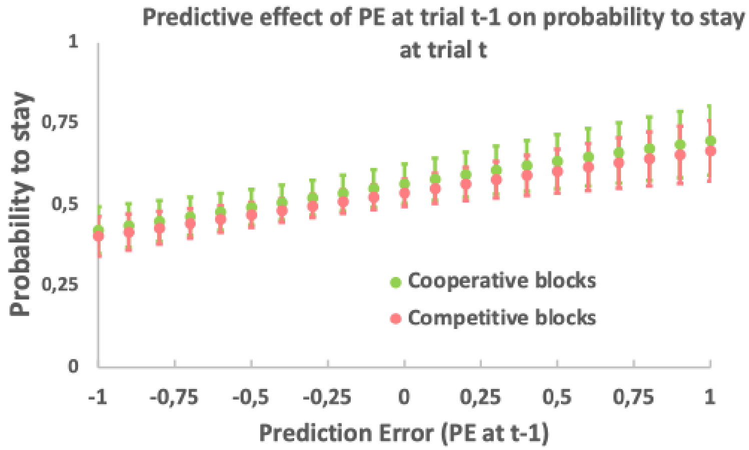

Supplementary figure 7. Predictive effect of the prediction error on the probability to stay do not depend on the block type (cooperative or competitive). Related to Computational models tracking intentions of the other agent in the Results section. Point represent the marginal effect in percentage of the prediction error on previous trial (t-1) on the probability to stay on the same target at trial t in Cooperative blocks (green) and in Competitive blocks (red) (e.g. for a -1 prediction error, the probability to stay in a trial classified as cooperative increase by 82%). Error bars are the 95% confidence interval. (random-effect logistic regression one-sided $\chi^2(1) = 0.04, p = 0.839$, not corrected for multiple comparison). N=31 independent participant each making 158 depend decision.

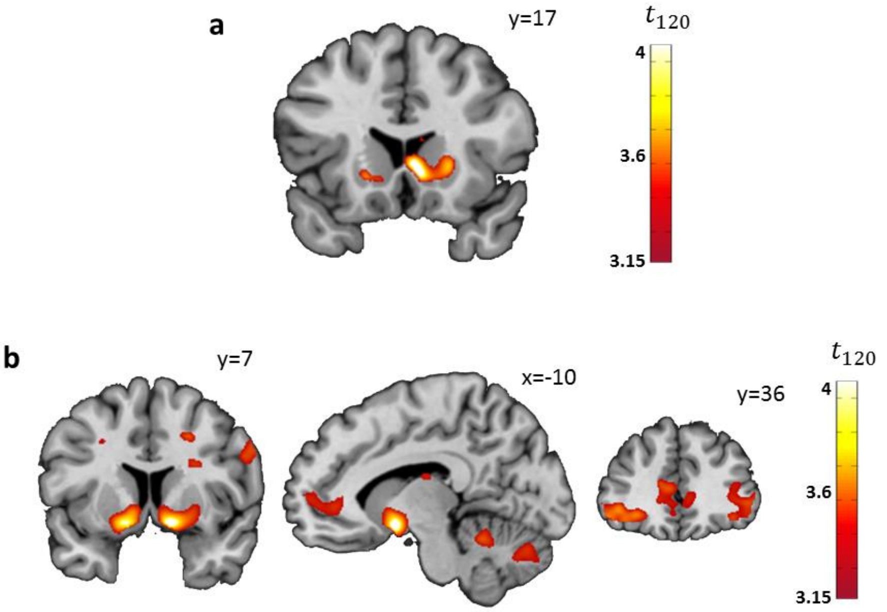

Supplementary figure 8. Common regions encoding Decision Value (top) and Prediction Error (bottom) for the Cooperative and Competitive experts. Related to Model-based analyses in the Results section. **a.** Common ventral striatal region encoding decision value of the cooperative and competitive experts. **b.** Common brain regions encoding prediction error of the cooperative and competitive experts included the ventral putamen, the anterior medial PFC, the posterior cingulate cortex and the lateral OFC.

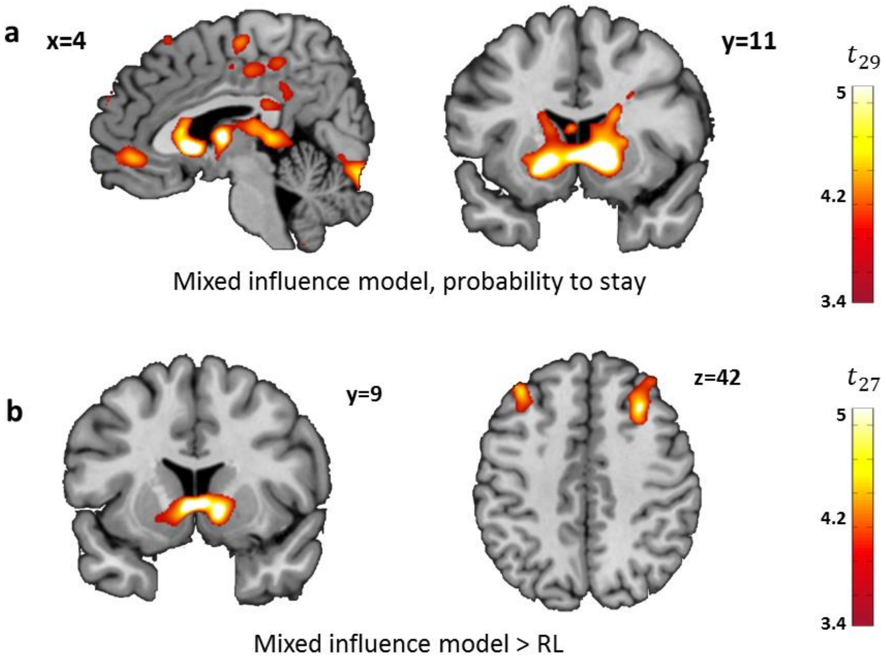

Supplementary figure 9. Decision Value of the Mixed-intentions Influence model correlates more with fMRI signal than Decision Value of the Reinforcement Learning model. Related to Model-based analyses in the Results section. **a.** Neural correlates of the decision value for staying on the same target as the previous trial, computed by the Mixed-intentions Influence model (Significant ventral striatum correlation x,y,z = 14,11,-2, $p < 0.05$ FWE corrected threshold at $p < 0.001$) **b.** Ventral Striatum (x,y,z = 6,12,0), bilateral dlPFC (x,y,z = -36, 33, 44 and x,y,z = 30,24,42) and Middle Temporal Gyrus (x,y,z = 65,-56,-8, $p < 0.05$ few, initial cluster forming threshold of $p < 0.001$) are best explained by the decision value for staying of the Mixed-intentions Influence model rather than the decision value for staying of a reinforcement learning model. See also **Supp. Note 2**.

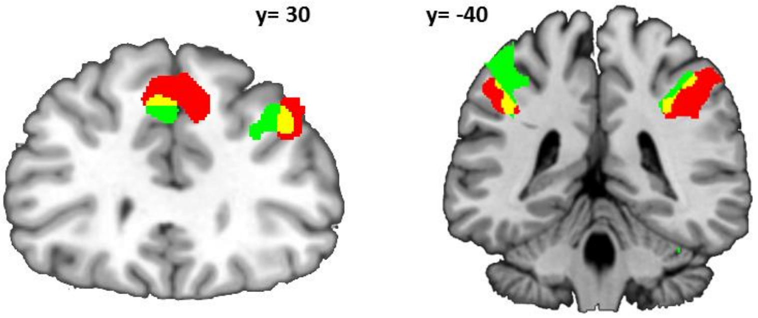

Supplementary figure 10. Overlap between regions encoding the reliability difference and regions encoding more PE during competitive trials. Related to fMRI results shown in Figures 5b and 6a in the results part. Overlap (in yellow) of brain regions correlating more with PE when trials are classified as competitive compared to those classified as cooperative (GLM2, in red), and of brain regions correlating with the controller at the time of choice (in green) ($p<0.005$ for display purpose).

References

1. Devaine, M., Hollard, G. & Daunizeau, J. The Social Bayesian Brain: Does Mentalizing Make a Difference When We Learn? *PLoS Comput. Biol.* **10**, (2014).

2. O'Doherty, J. *et al.* Why and how the brain weights contributions from a mixture of experts. 1–18 (2021). doi:10.31234/osf.io/ns6kq

3. Wan Lee, S., Shimojo, S. & O'Doherty, J. P. Neural Computations Underlying Arbitration between Model-Based and Model-free Learning. *Neuron* **81**, 687–699 (2014).

4. Charpentier, C. J., Iigaya, K. & O'Doherty, J. P. A Neuro-computational Account of Arbitration between Choice Imitation and Goal Emulation during Human Observational Learning. *Neuron* **106**, 687-699.e7 (2020).

5. Friston, K. *et al.* Active inference and learning. *Neurosci. Biobehav. Rev.* **68**, 862–879 (2016).

6. Mathys, C., Daunizeau, J., Friston, K. J. & Stephan, K. E. A Bayesian foundation for individual learning under uncertainty. *Front. Hum. Neurosci.* **5**, 1–20 (2011).

7. Mathys, C. D. *et al.* Uncertainty in perception and the Hierarchical Gaussian Filter. *Front. Hum. Neurosci.* **8**, 1–24 (2014).

8. Brodersen, K. H. *et al.* Variational Bayesian mixed-effects inference for classification studies. *Neuroimage* **76**, 345–361 (2013).
