# Supplementary material for: Neurocomputational mechanisms involved in adaptation to fluctuating intentions of others
Source: Nat Commun. 2024 Apr 12;15:3189. doi: 10.1038/s41467-024-47491-2 (PMC11014977; doi:10.1038/s41467-024-47491-2)
Supplement: Supplementary file 3 — Reporting Summary [file 41467_2024_47491_MOESM3_ESM.pdf]

nature portfolio

Corresponding author(s): Jean-Claude DREHER

Last updated by author(s): 28/11/23

Reporting Summary

Nature Portfolio wishes to improve the reproducibility of the work that we publish. This form provides structure for consistency and transparency in reporting. For further information on Nature Portfolio policies, see our Editorial Policies and the Editorial Policy Checklist.

Statistics

For all statistical analyses, confirm that the following items are present in the figure legend, table legend, main text, or Methods section.

n/a	Confirmed	
☐	☒	The exact sample size (n) for each experimental group/condition, given as a discrete number and unit of measurement
☐	☒	A statement on whether measurements were taken from distinct samples or whether the same sample was measured repeatedly
☐	☒	The statistical test(s) used AND whether they are one- or two-sided<br>*Only common tests should be described solely by name; describe more complex techniques in the Methods section.*
☐	☒	A description of all covariates tested
☐	☒	A description of any assumptions or corrections, such as tests of normality and adjustment for multiple comparisons
☐	☒	A full description of the statistical parameters including central tendency (e.g. means) or other basic estimates (e.g. regression coefficient) AND variation (e.g. standard deviation) or associated estimates of uncertainty (e.g. confidence intervals)
☐	☒	For null hypothesis testing, the test statistic (e.g. F, t, r) with confidence intervals, effect sizes, degrees of freedom and P value noted<br>*Give P values as exact values whenever suitable.*
☐	☒	For Bayesian analysis, information on the choice of priors and Markov chain Monte Carlo settings
☐	☒	For hierarchical and complex designs, identification of the appropriate level for tests and full reporting of outcomes
☐	☒	Estimates of effect sizes (e.g. Cohen's d, Pearson's r), indicating how they were calculated

Our web collection on statistics for biologists contains articles on many of the points above.

Software and code

Policy information about availability of computer code

Data collection	Brain Imaging was performed on a 3T Siemens Magnetom Prisma MRI scanner. The stimuli presentation was made using Presentation software (14.0).
Data analysis	Behavioral data was analyzed using STATA 14.1. The computational modeling was performed using Matlab 2015b. Brain imaging data were analyzed using SPM12 in combination with Matlab 2015b. The GitHub codes developed in the study are accessible at:<br>https://github.com/remiphilipp/Mixture_intention.git<br>DOI: 10.5281/zenodo.10299140

For manuscripts utilizing custom algorithms or software that are central to the research but not yet described in published literature, software must be made available to editors and reviewers. We strongly encourage code deposition in a community repository (e.g. GitHub). See the Nature Portfolio guidelines for submitting code & software for further information.

Data

Policy information about availability of data

All manuscripts must include a data availability statement. This statement should provide the following information, where applicable:

- Accession codes, unique identifiers, or web links for publicly available datasets
- A description of any restrictions on data availability
- For clinical datasets or third party data, please ensure that the statement adheres to our policy

Behavioral data and Pre-processed fMRI are available at:
DOI: 10.5281/zenodo.10299140

https://neurovault.org/collections/EOCXPHRJ/

Research involving human participants, their data, or biological material

Policy information about studies with human participants or human data. See also policy information about sex, gender (identity/presentation), and sexual orientation and race, ethnicity and racism.

Reporting on sex and gender	Sex and gender were not considered in the study design. The findings apply to both sex (men and women). Sex was determined based on self reporting. Information about gender has not been collected. Sex -based analyses were not performed because the n is relatively low to perform such analysis (n=31, 17 women).
Reporting on race, ethnicity, or other socially relevant groupings	No socially constructed or relevant categorization variable was used in the manuscript.
Population characteristics	age 20-40, M = 27, SD = 5.1
Recruitment	Participants were recruited via a daily local newspaper and the University of Lyon 1 mailing list. All participants were screened to exclude those with medical conditions including psychological or physical illnesses or a history of head injury..
Ethics oversight	National Ethics Committee (CPP Est II: 18/592, ANSM: 2018-A01135-50)

Note that full information on the approval of the study protocol must also be provided in the manuscript.

Field-specific reporting

Please select the one below that is the best fit for your research. If you are not sure, read the appropriate sections before making your selection.

☒ Life sciences ☐ Behavioural & social sciences ☐ Ecological, evolutionary & environmental sciences

For a reference copy of the document with all sections, see nature.com/documents/nr-reporting-summary-flat.pdf

Life sciences study design

All studies must disclose on these points even when the disclosure is negative.

Sample size	No statistical method was used to predetermine sample size. We simply used a sample size greater than the norm in the field (n=31> 20). Moreover, our results are not dependent on individual differences across participants.
Data exclusions	Ali recruted participants were analyzed.
Replication	Replication of model simulation was successfully done at least 3 times. No direct replication of the experiment was perform.
Randomization	Subjects were not allocated to different treatment groups. Ali experimental conditions were presented interleaved. Location of stimuli was randomized.
Blinding	The within-subject design that we used in this study did not require blinding.

Reporting for specific materials, systems and methods

We require information from authors about some types of materials, experimental systems and methods used in many studies. Here, indicate whether each material, system or method listed is relevant to your study. If you are not sure if a list item applies to your research, read the appropriate section before selecting a response.

Materials & experimental systems

n/a	Involved in the study
☒	☐ Antibodies
☒	☐ Eukaryotic cell lines
☒	☐ Palaeontology and archaeology
☒	☐ Animals and other organisms
☒	☐ Clinical data
☒	☐ Dual use research of concern
☒	☐ Plants

Methods

n/a	Involved in the study
☒	☐ ChIP-seq
☒	☐ Flow cytometry
☐	☒ MRI-based neuroimaging

Magnetic resonance imaging

Experimental design

Design type: Task, Event-related.

Design specifications: Each subject completed 2 runs with 84 and 79 trials respectively. Blocks of 13 trials and 10 trials in each condition were interleaved. The jitter between decision and outcome was in 3 to 5 seconds and inter trial interval was in 1.5 to 3.5 seconds.

Behavioral performance measures: On each trial, 1) a binary decision whether to choosen right target or not (choose the left target), 2) reaction time, 3) a binary indicator of the participant's outcome.

Acquisition

Imaging type(s): Tl-weighted, T2*-weighted gradient-echo planar imaging, Field map

Field strength: 3T

Sequence & imaging parameters: Single-shot EPI, TR /TE= 1600/30, flip angle 75', multiband acquisition (accelerator factor of 2), in an ascending interleaved manner with slices interlaced 2.40 mm thickness, FOV = 210 mm. We also use the iPAT mode with an accelerator factor of 2 and the GRAPPA method reconstruction. The number of volumes acquired varied given the time the participant took to make their decisions. The first acquisition was made after stabilization of the signal. Whole-brain high-resolution Tl-weighted structural scans (0.8 x 0.8 x 0.8 mm)

Area of acquisition: Whole-brain

Diffusion MRI: ☐ Used ☒ Not used

Preprocessing

Preprocessing software: SPM12

Normalization: Each structural image was segmented into gray matter, white matter and cerebral spinal fluid images using a nonlinear deformation field and mapped on a template. The deformations were further applied to both structural and functional images to create new images spatially normalized to Montreal Neurological Institute (MNI) space.

Normalization template: SPM12 MNI template

Noise and artifact removal: 6 motion correction parameters were estimated from the realignment procedure and were entered as nuisance covariates. The onset time of any button press was entered as stick function to rem ove the potential motion effects. Artrepair was used after preprocessing to de-weight the scan if the movements were too large. We also de-weighted scan with too high or low intensity compare to the mean intensity of scans of the session.

Volume censoring: If variation of intensity was higher than 1.3% from the mean or if motion was higher than 0.5 mm between 2 TR, scans were de-weighted.

Statistical modeling & inference

Model type and settings: Mass univariate; fixed-effects within subject to combine fMRI data across runs; random-effects across subjects for second-level analyses.

Effect(s) tested: The group level effects was tested with one-sample or two sample t-test using SPM12. The statistical inference was conducted using Gaussian random field theory as implemented in SPM12 to obtain clusters satisfying P<0.05, family-wise error (FWE) corrected at a cluster-defining threshold of P<0.001 uncorrected.

Specify type of analysis: ☒ Whole brain ☐ ROI-based ☐ Both

Statistic type for inference
(See Eklund et al. 2016)

Voxel-wise inference

Correction

The clusters satisfying P<0.05, family-wise error (FWE) corrected at a cluster-defining threshold of P<0.001 uncorrected.

Models & analysis

n/a	Involved in the study
☐	☒ Functional and/or effective connectivity
☐	☐ Graph analysis
☐	☐ Multivariate modeling or predictive analysis

Functional and/or effective connectivity

The generalized Psycho-Physiological Interaction (gPPI) was conducted using the Conn Toolbox

Graph analysis

N/A

Multivariate modeling and predictive analysis

Multivariate Modeling :
Independent variables of the behavioral analyses (at t-1 t-2 and t-3 for all variables): Previous winning, Switch, Cooperativity signature (WSLS), and real mode of interaction.
Training: with the VBA toolbox ("Variational Bayesian Analysis"),
see: https://mbb-team.github.io/VBA-toolbox/
Evaluation metrics: Free Energy (Karl Friston).
Computational models are described in details in the methods section.
